# Exploring narcissism and human- and animal-centered empathy in pet owners

**DOI:** 10.3389/fpsyg.2023.1087049

**Published:** 2023-03-30

**Authors:** Miranda Giacomin, Emma E. Johnston, Eric L. G. Legge

**Affiliations:** Department of Psychology, MacEwan University, Edmonton, AB, Canada

**Keywords:** grandiose narcissism, vulnerable narcissism, pet ownership, human-centered empathy, animal-centered empathy

## Abstract

Having empathy for others is typically generalized to having empathy for animals. However, empathy for humans and for animals are only weakly correlated. Thus, some individuals may have low human-centered empathy but have high animal-centered empathy. Here, we explore whether pet owners who are high in narcissism display empathy towards animals despite their low human-centered empathy. We assessed pet owners’ (*N* = 259) three components of trait narcissism (Agentic Extraversion, Antagonism, and Narcissistic Neuroticism), human- and animal-centered empathy, attitudes towards animals, and their pet attachment. We found that Agentic Extraversion was unrelated to both human- and animal-centered empathy. We also found that Antagonism was related to less empathy for both humans and animals, as well as more negative attitudes towards animals. Lastly, we found that Narcissistic Neuroticism was unrelated to human-centered empathy and positively related to animal-centered empathy and attitudes towards animals. This research furthers our understanding of the relation between empathy towards humans and animals and provides insight into whether animal-assisted approaches may be useful for empathy training in those with narcissistic characteristics.

## Introduction

Empathetic individuals are often characterized by their benevolent nature and caring demeanor (Davis, 1983; Bloom, 2017). In contrast, individuals with narcissistic traits are characterized by a lack of regard for others and their self-serving attitudes (Miller et al., 2011; Back et al., 2013; Crowe et al., 2018; Weiss et al., 2019). Although individuals high in narcissism tend to display less empathy towards humans, there is limited research exploring how narcissism relates to animal-centered empathy and attitudes towards animals among pet owners. Given that the core of both grandiose and vulnerable narcissism is relatively low empathy (Hart et al., 2018; Urbonaviciute and Hepper, 2020; Simard et al., 2022), it is important to explore whether this empathy deficit is directed towards both humans *and* animals.

### Pet ownership and empathy

Empathy is a vicarious emotional response to others’ emotional experiences and includes feeling sympathy and concern for others (Mehrabian and Epstein, 1972; Davis, 1983; Paul, 2000). It is widely believed that individuals who are highly empathetic towards humans will exhibit that same level of empathy towards animals (i.e., experience a vicarious emotional response to the emotions of animals; Eisenberg, 1988; Paul, 2000). For instance, individuals who are violent towards animals and have low animal-centered empathy may display the same aggression and low empathy towards humans (Schwartz et al., 2012). Although human- and animal-centered empathy tap the same psychological mechanisms, they are independent and influenced by separate factors (Paul, 2000). For example, Gómez-Leal et al. (2021) revealed that individuals who owned a pet display higher animal-centered empathy, but lower human-centered empathy, compared to those who have not owned a pet. Likewise, human- and animal-centered empathy were related among individuals who had adopted a pet but not among those who had relinquished a pet (Cardoso et al., 2022). Thus, human-centered empathy may be related to, but distinct from, animal centered-empathy.

In addition, there are conflicting views on the relation between holding positive attitudes towards animals and human-centered empathy. On the one hand, some researchers have argued that having higher human-centered empathy relates to greater concern for animal welfare (Taylor and Signal, 2005). On the other hand, researchers have demonstrated low human-centered empathy relates to enhanced concern for animal welfare. For example, some militant animal activists exhibit a great sense of animal-centered empathy and concern for animal welfare while directing violence towards humans in the name of their cause (Paton, 1993; Amiot and Bastian, 2015). Here, we explore the possibility that the extent to which someone differs in the amount of human- vs. animal-centered empathy may be impacted by their personality traits, namely their narcissistic tendencies.

### Narcissism

**Figure 1:**
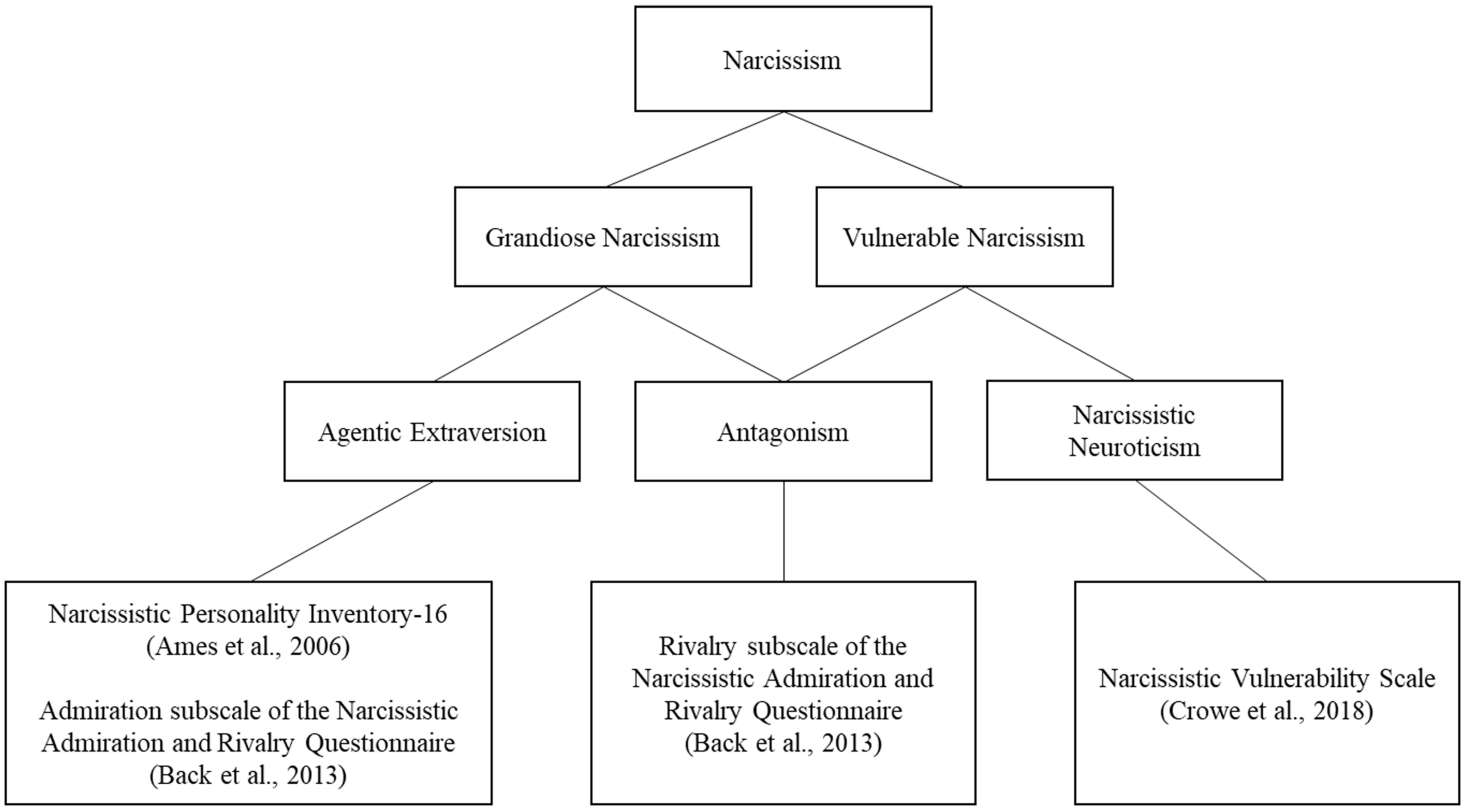
A representation of the Trifurcated Model of Narcissism and the assessments used to measure each of the three factors of narcissism in the present study.

Narcissism^1^ is broadly distinguished into two prototypical dimensions (e.g., Miller et al., 2021). Grandiose narcissism is characterized by a need for attention and admiration and a strong sense of self-importance, whereas vulnerable narcissism is characterized by introversion, hypersensitivity, defensiveness, withdrawal, and negative affect (e.g., anxiety, shame; Miller et al., 2016). Both dimensions of narcissism share similar qualities such as self-absorption, interpersonal antagonism, entitlement, and low human-centered empathy (Miller et al., 2016; Crowe et al., 2018; Weiss et al., 2019). More recently, researchers have argued for a Trifurcated Model of Narcissism, suggesting narcissism can be divided into three facets: Agentic Extraversion, Narcissistic Neuroticism, and Antagonism (see Figure 1; Crowe et al., 2018; Krizan and Herlach, 2018; Weiss et al., 2019; Miller et al., 2021). Agentic Extraversion (also labeled admiration or grandiosity) is uniquely associated with grandiose narcissism. Individuals high in agentic extraversion are assertive, dominant, attention seeking, and high in self-esteem. Narcissistic Neuroticism (also labeled vulnerability) is uniquely associated with vulnerable narcissism. Individuals high in narcissistic neuroticism are hypersensitive, struggle to regulate their emotions, and are low in self-esteem. Antagonism (also labeled rivalry or entitlement) lies at the core of both grandiose and vulnerable narcissism (Weiss et al., 2019). Individuals high in Antagonism have a hostile interpersonal orientation, where they are callous, deceitful, exploitive, and low in empathy.

Researchers have found that exploring narcissism at the trifurcated level is helpful for highlighting narcissism’s relations with important outcomes (e.g., Edershile and Wright, 2021; Mahadevan and Jordan, 2022; Szymczak et al., 2022). In a recent meta-analysis, Simard et al. (2022) found that each narcissistic trait can account for differences in empathic responding toward humans. Agentic Extraversion, Antagonism, and Narcissistic Neuroticism were related to less human-centered empathic concern, yet only Narcissistic Neuroticism predicted greater feelings of personal distress in response to another person’s suffering (Simard et al., 2022). Notably, the three-factor model of narcissism can provide insight into the motives that might predict differences in empathic responding. For example, individuals high in Agentic Extraversion may display lower empathy due to a lack of concern for others or less consideration for the perspective of others, while individuals high in Narcissistic Neuroticism may display lower empathy due to their overwhelming insecurities, and individuals high in Antagonism may have more pathological and antisocial motives for their empathy deficit (Simard et al., 2022). Thus, adopting the three-factor model of narcissism is useful for understanding an individual’s empathy profile; however, limited research has explored how narcissism may relate to animal-centered empathy, attitudes towards animals, and pet attachment.

### Narcissism and pet ownership

Given that the core of both grandiose and vulnerable narcissism is Antagonism (Hart et al., 2018; Urbonaviciute and Hepper, 2020), it is important to explore whether individuals high (vs. low) in narcissism express lower empathic concern towards humans *and* animals. To date, researchers have primarily focused on the personality traits of pet owners, but have not found that pet owners versus non-pet owners differ in terms of their narcissistic traits or self-esteem (Johnson and Rule, 1991; Fraser et al., 2020). However, pet owners may have pets for a host of reasons, including status and control or love and companionship (Beverland et al., 2008). The Extended Agency Model of narcissism (Campbell and Foster, 2007) argues that individuals high in narcissism are particularly motivated by agentic rewards like attaining status or power and are less motivated by communal rewards like forming close relationships with people and (possibly) animals. Thus, an individual’s narcissistic traits may predict their degree of animal-centered empathy, their attitudes towards animals, and their pet attachment.

Individuals high in grandiose narcissism prefer to be seen as superior and dominant (Campbell and Foster, 2007). As a result, individuals high in grandiose narcissism might be motivated to own a pet to feel a sense of power over another living being (Alba and Haslam, 2015). Researchers have found a positive relation between being high in dominance and owning a dog (Alba and Haslam, 2015) and individuals high (vs. low) in grandiose narcissism report being more attached to traditional pets (e.g., dog, cat; Vonk et al., 2016). Despite being more attached to their pet, individuals high (vs. low) in grandiose narcissism report more negative attitudes towards animals, but not more acts of cruelty towards animals (Kavanagh et al., 2013). Holding more negative attitudes towards animals, however, predicts lower animal-centered empathy (Ellingsen et al., 2010). Thus, individuals who score high (vs. low) in grandiose narcissism, particularly Antagonism, may own a pet to fulfill their desire of dominance and superiority over a living being rather than for love and companionship, and thereby display less empathy towards animals.

Alternatively, individuals high in grandiose narcissism, especially those high in Agentic Extraversion, tend to thrive in competitive scenarios where they directly compare themselves to others. To regulate their grandiose sense of self, narcissists seek attention from others to self-enhance (Campbell and Foster, 2007; Back et al., 2013). Given individuals high in grandiose narcissism focus on deriving self-esteem from others’ approval, it may be that they have low empathy towards humans but not animals, as animals pose little competition for them.

Individuals high (vs. low) in vulnerable narcissism often fear rejection, which may lead them to avoid social relationships (Dickinson and Pincus, 2003), and, in turn, feel increasingly lonely (Çağlayaner, and Çoklar-Okutkan, 2021). Researchers have found that owning a pet can satisfy social needs, reduce the psychological distress that results from social rejection, and reduce loneliness (Staats et al., 2008; McConnell et al., 2011). Paul et al. (2014) demonstrates that pet owners who feel socially disconnected and have high interpersonal sensitivity report a greater tendency to turn to their pets for support. Indeed, individuals high (vs. low) in vulnerable narcissism tend to be more attached to their non-traditional pets (e.g., parrot, reptile, nonhuman primate, and barnyard animal; Vonk et al., 2016). Like human attachment, animal attachment involves fulfilling a special friendship, providing a secure base, and creating an affectionate bond with another living being (Hawkins et al., 2017). Attachment to a pet predicts an individual’s attitude towards animals, such that those with a greater sense of attachment to their pet have greater concern for animal welfare (Hawkins et al., 2017). Moreover, having more positive attitudes towards animals correlates with higher animal-centered empathy (Ellingsen et al., 2010). Thus, owning a pet may be beneficial for individuals high in vulnerable narcissism because it may fulfill their social needs, reduce loneliness, and enhance social support, all without concern of rejection, as the animal’s support is seen as unconditional (McNicholas and Collis, 1995; Fine and Beck, 2015). As such, pet owners who are high in Narcissistic Neuroticism, a trait uniquely associated with vulnerable narcissism (relative to other forms), may report greater animal-centered empathy, more positive attitudes towards animals, and higher pet attachment compared to those who are low in Narcissistic Neuroticism.

### The current study

Although previous research has focused on the personality traits of pet owners, little research has explored how personality traits may separately impact animal- and human-centered empathy. To our knowledge, there is no research that has explored the relation between the three aspects of narcissism (i.e., Agentic Extraversion, Narcissistic Neuroticism, and Antagonism) and human- and animal-centered empathy, attitudes towards animals, and pet attachment among pet owners. Adopting a multi-faceted exploration of people’s narcissistic tendencies allows researchers to better understand how narcissistic traits impact people’s relationships with humans and animals.

Here, we explore the relation between narcissism and human- and animal-centered empathy among pet owners. We also explore how narcissistic traits relate to pet attachment and people’s attitudes towards animals. The project method was pre-registered.^2^ We hypothesized that grandiose narcissism and vulnerable narcissism will be negatively correlated with human-centered empathy and positively correlated with animal-centered empathy (pre-registered). More specifically, we predict that Agentic Extraversion, Antagonism, and Narcissistic Neuroticism will be associated with lower human-centered empathy (Back et al., 2013; Crowe et al., 2018). Given that animals may be a status-symbol for narcissists, we expect that Agentic Extraversion and Narcissistic Neuroticism may be positively related to empathy for animals, but Antagonism will be negatively related to empathy for animals.

In addition, we explore the relation between narcissism and attitudes towards animals and pet attachment. We expect that Antagonism will predict more negative attitudes towards animals, Narcissistic Neuroticism will predict more positive attitudes towards animals, and Agentic Extraversion will be unrelated to attitudes towards animals. We also predict a negative correlation between grandiose and vulnerable narcissism and pet attachment (pre-registered). More specifically, individuals high (vs. low) in Antagonism tend to care less about others and thus may report less attachment to their pets. Individuals high (vs. low) in Agentic Extraversion and Narcissistic Neuroticism may report greater pet attachment.

## Method

### Participants

Two hundred and eighty four undergraduate university students (205 female, 73 male, 6 missing; *M*_age_ = 21.46 years, SD = 5.61) completed the study in exchange for partial course credit. Because past research has shown that those who own both a cat and a dog are more empathetic than those who own only a dog, cat, or neither (Daly and Morton, 2006), recruitment for the current study was restricted to only those who indicated that they own both a cat and a dog during a pre-screening questionnaire (i.e., participants answered yes to “Do you own a dog?’ and “Do you own a cat?”). Approximately 12% of our convenience sample indicated that they owned both a dog and a cat. As such, it is important to caution that this sample may not be representative of the general population. An *a priori* power analysis indicated that 197 participants would provide 95% power for a two-tailed small to medium correlation (effect size |*ρ*| = 0.25; *α* = 0.05). Because larger sample sizes allow for more accurate estimates, we indicated in our pre-registration that we would terminate data collection at 300 participants or the end of the academic semester. Finally, we excluded participants who indicated they did not currently own a pet (*n* = 2), those who scored +/− 3 standard deviations on our primary variables (*n* = 16), and those who completed 90% of the study or less (*n* = 6), leaving a final sample of 259 participants (191 female, 63 male, 5 missing; *M*_age_ = 21.42 years, SD = 5.48).

### Materials

#### Narcissism

Participants completed three measures of narcissism that assessed Agentic Extraversion, Antagonism, and Narcissistic Neuroticism (see Figure 1).

To assess Agentic Extraversion, participants completed the Narcissistic Personality Inventory-16 (NPI-16; Ames et al., 2006) and the Admiration subscale of the Narcissistic Admiration and Rivalry Questionnaire (NARQ; Back et al., 2013). The NPI-16 asks participants to select one of two options, one of which is more indicative of narcissism (e.g., “I find it easy to manipulate people”). Scores were computed by averaging the number of narcissistic options selected (*M* = 0.18, SD = 0.17, *α* = 0.69), such that high scores indicate higher grandiose narcissism. The Admiration subscale of the NARQ includes nine items assessing narcissistic admiration (e.g., “I am great”) rated on a scale from 1 (*not agree at all*) to 6 (*agree completely*), which were averaged such that higher scores indicate higher admiration (*M* = 3.27, SD = 0.73, *α* = 0.79). Both measures were highly correlated (*r* = 0.59, *p* < 0.001), so we standardized scores and calculated an average score for Agentic Extraversion.

To assess Antagonism, participants completed the Rivalry subscale of the NARQ, which includes nine items assessing narcissistic rivalry (e.g., “I secretly take pleasure in the failure of my rivals”) rated on a scale from 1 (*not agree at all*) to 6 (*agree completely*). The scores were averaged such that higher scores indicate higher rivalry (*M* = 2.00, SD = 0.65, *α* = 0.80).

To assess Narcissistic Neuroticism, participants completed the 12-item Narcissistic Vulnerability Scale (NVS; Crowe et al., 2018). Participants were instructed to rate how much they identify with 12 adjectives (e.g., “underappreciated,” “fragile”) on a scale of 1 (*not at all*) to 7 (*extremely*). Responses were averaged (*M* = 2.95, SD = 0.91, *α* = 0.82), such that higher scores indicate higher vulnerable narcissism.

#### Human-centered empathy

Human-centered empathy was measured using the Interpersonal Reactivity Index (IRI; Davis, 1983) and the Questionnaire for Measures of Emotional Empathy (QMEE; Mehrabian and Epstein, 1972). The IRI is a 28-item self-report scale composed of four subscales: empathic concern (EC-IRI; e.g., “When I see someone being taken advantage of, I feel kind of protective towards them”), fantasy (FS-IRI; e.g., “I really get involved with the feelings of the characters in a novel”), personal distress (PD-IRI; e.g., “I sometimes feel helpless when I am in the middle of a very emotional situation”), and perspective taking (PT-IRI; e.g., “I try to look at everybody’s side of a disagreement before I make a decision”). Each item uses a five-point Likert scale ranging from 1 (*does not describe me at all*) to 5 (*describes me very well*). Recent research exploring the factor structure of the IRI determined that the most reasonable assessment of empathic responding is to average scores on the empathic concern (*M* = 4.08, SD = 0.58, *α* = 0.74) and perspective taking subscales (*M* = 3.61, SD = 0.70, *α* = 0.79), such that higher scores indicate greater empathic responding (Wang et al., 2020).

The QMEE consists of 33-items that measure affective role-taking empathy (e.g., “The people around me have a great influence on my mood”). Responses were recorded using a five-point Likert scale from 1 (*strongly disagree*) to 5 (*strongly agree*). We computed a mean score (*M* = 3.78, SD = 0.70, *α* = 0.82), such that higher scores indicate higher affective role-taking empathy.

Both human empathy measures were highly correlated (*r* = 0.61, *p* < 0.001), so we standardized scores and computed an average score for human-centered empathy.

#### Animal-centered empathy

Participants’ animal-centered empathy was measured using the Animal Empathy Scale (AES; Paul, 2000), which is an adaptation of the QMEE. The AES is a 22-item self-report questionnaire that uses a scale ranging from 1 (*strongly disagree*) to 9 (*strongly agree*) to assess participants’ feelings towards animals and the treatment of animals (e.g., “Seeing animals in pain upsets me”). Scores were averaged (*M* = 7.03, SD = 0.87, *α* = 0.82), such that higher scores indicate higher animal-centered empathy.

#### Attitudes and attachment toward animals

Two scales that may influence animal-centered empathy (Paul, 2000) were also included: the Pet Attachment and Life Impacts Scale (PALS; Cromer and Barlow, 2013) and the Animal Attitude Scale (AAS; Herzog et al., 2015). The PALS is used to assess participants’ attachment to pets, the positive and negative aspects of their relationships with pets, and the impact that their pet has on them. If participants live with more than one pet, they were instructed to respond with their favorite pet in mind. This questionnaire consists of 39 items with four factors that measure love (e.g., “My pet gives me unconditional love”), regulation of emotions (e.g., “My pet teaches me to trust”), personal growth (e.g., “Having a pet has helped my health”), and negative impact (e.g., “Having a pet is stressful”; the four negative impact items were reverse scored). Participants recorded their response using a scale from 1 (*not at all*) to 5 (*very much*). Items in each subscale are averaged such that higher scores indicate that their pets provide more love (*M* = 4.40, SD = 0.52, *α* = 0.89), regulation of emotions (*M* = 3.77, SD = 0.95, *α* = 0.90), and personal growth (*M* = 3.99, SD = 0.78, *α* = 0.75), as well as having less of a negative impact on their life (*M* = 4.38, SD = 0.47, *α* = 0.44).

The AAS was used to assess participants’ attitude towards animals and utilized a scale from 1 (*strongly disagree*) to 5 (*strongly agree*) to rate 10 items (e.g., “It is morally wrong to hunt wild animals just for sport”). We averaged participants’ responses (*M* = 3.74, SD = 0.57, *α* = 0.77), such that higher scores indicate a stronger concern for animal welfare.

#### Demographics

The demographic questionnaire assessed participants’ age, gender, ethnicity, and pet ownership history. In our analyses, we control for gender because women tend to have higher human- and animal-centered empathy than men (Paul, 2000; Klein and Hodges, 2001; Serpell, 2004; Angantyr et al., 2011), men tend to be higher in narcissism than women (Grijalva et al., 2015; Weidmann et al., 2023), and gender may be associated with differences in animal and pet attachment (Vonk et al., 2016; pre-registered). Participants were asked questions about their pet ownership history [e.g., “Have you previously owned a dog (cat)?”]. Both past and current ownership of pets have been associated with higher animal-centered empathy (Paul, 2000; Bagley and Gonsman, 2005). The majority of our sample indicated previously owning a pet (*n* = 243). We also asked participants about the extent to which they care for their current pet. The majority of participants (*n* = 165) indicated that they considered themselves to be the primary caregiver for their pet. Of those who indicated they were the primary caregiver, 162 of them said they have significant active engagement (i.e., feeding, cuddling, and walking) with their pet.

We had also asked participants about whether they grew up with siblings, lived with children (12 years or younger), were a parent or guardian for a child, or are involved in caring for children because caring for children has been associated with higher human-centered empathy (Paul, 2000). Most of our participants indicated they grew up with siblings (*n* = 221) and only a few participants reported living with a child (*n* = 42), being a parent/guardian (*n* = 16) or having a job that involves the frequent care of children (*n* = 30). We also asked participants whether they had a job involving the frequent care of animals because animal protection workers report higher human-centered empathy and more positive attitudes toward animals compared to members of the general community (Signal and Taylor, 2007); however, only nine participants reported working with animals. Given the lack of variability in the sample, we do not explore these factors as potential covariates any further.

### Procedure

After providing their informed consent, participants completed a demographics questionnaire. Questionnaires were assembled into three groups—Narcissism (i.e., NARQ, NPI-16, NVS), human-centered empathy (i.e., QMEE, IRI), and animal-centered Empathy (i.e., AES, PALS, AAS)—and were presented to participants in random order.

## Results

Because our measures used different response scales, we standardized all variables by transforming them to z-scores prior to analysis. We used bivariate correlations to explore the relation between narcissism and animal- and human-centered empathy, attitudes towards animals, and pet attachment in pet owners (see Table 1). Agentic Extraversion was unrelated to both human and animal-centered empathy, attitudes towards animals, and pet attachment. Antagonism was negatively related to both human and animal-centered empathy, attitudes towards animals, and experiencing negative impacts from pets. Narcissistic Neuroticism was unrelated to human and animal-centered empathy but positively predicted more positive attitudes towards animals, greater negative impacts from owning a pet, and more emotion regulation from pet ownership.

**Table 1:** Zero-order correlations between narcissism and animal- and human-centered empathy, pet attachment, and attitude towards animals.

Variable	1	2	3	4	5	6	7	8	9
1. Agentic Extraversion	-								
2. Antagonism	0.31^**^	-							
3. Narcissistic Neuroticism	−0.02	0.40^**^	-						
4. Human-centered empathy	−0.06	−0.35^**^	−0.00	-					
5. Animal-centered empathy	−0.06	−0.26^**^	0.11	0.59^*^	-				
6. Attitudes towards animal	−0.04	−0.18^**^	0.17^**^	0.31^**^	0.57^**^	-			
7. Emotion regulation—pet attachment	−0.01	−0.08	0.15^*^	0.33^**^	0.48^**^	0.32^**^	-		
8. Love—pet attachment	0.04	−0.10	0.05	0.30^**^	0.51^**^	0.35^**^	0.76^**^	-	
9. Negative impact—pet attachment	−0.05	−0.19^**^	−0.16^**^	0.13^*^	0.12	0.07	0.00	−0.01	-
10. Personal growth—pet attachment	−0.01	−0.05	0.09	0.31^**^	0.44^**^	0.22^**^	0.72^**^	0.68^**^	−0.10

^*^p < 0.05, ^**^p < 0.01.

### Narcissism, attitude towards animals, and animal attachment

**Figure 2:**
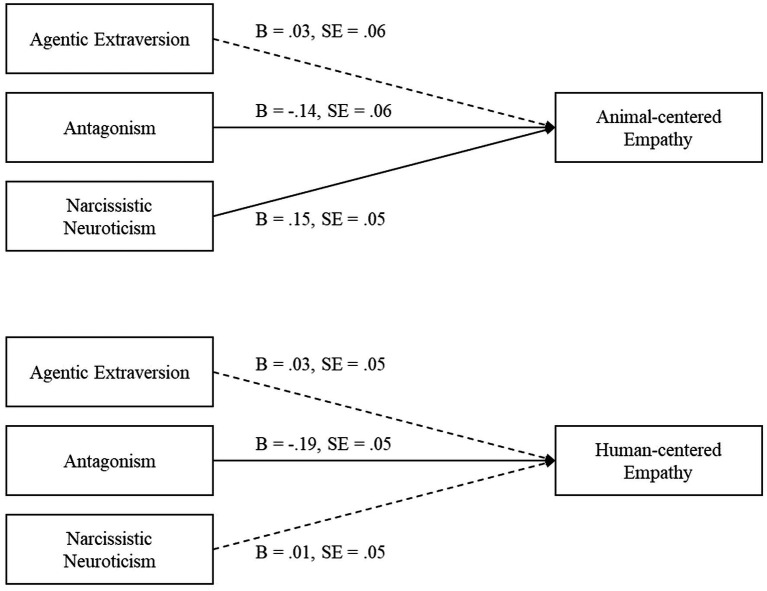
A summary of the regression analyses where Agentic Extraversion, Antagonism, and Narcissistic Neuroticism predict animal-centered empathy and human-centered empathy, controlling for gender, human-centered empathy, and animal-centered empathy, respectively. Solid lines indicate significant pathways.

We conducted a series of regression analyses to examine whether Agentic Extraversion, Antagonism, and Narcissistic Neuroticism predicts animal and human-centered empathy, respectively (see Figure 2; for the full regression table, see Supplementary Materials: Supplementary Table 1). We control for gender (coded as 1 = male, 0 = female) in our regressions.

First, we used Agentic Extraversion, Antagonism, and Narcissistic Neuroticism to predict human-centered empathy, controlling for animal-centered empathy, *B* = 0.40, SE = 0.05, *t*(248) = 8.48, *p* < 0.001, 95% CI [0.31, 0.49], and gender, *B* = −0.18, SE = 0.10, *t*(248) = −1.86, *p* = 0.064, 95% CI [−0.37, 0.01]. Agentic Extraversion, *B* = 0.03, SE = 0.05, *t*(248) = 0.58, *p* = 0.584, 95% CI [−0.07, 0.12], and Narcissistic Neuroticism did not predict human-centered empathy, *B* = 0.01, SE = 0.05, *t*(248) = 0.15, *p* = 0.879, 95% CI [−0.08, 0.10]. As expected, Antagonism negatively predicted human-centered empathy, *B* = −0.19, SE = 0.05, *t*(248) = −3.77, *p* < 0.001, 95% CI [−0.28, −0.09].

Next, we used Agentic Extraversion, Antagonism, and Narcissistic Neuroticism to predict animal-centered empathy while controlling for human-centered empathy, *B* = 0.56, SE = 0.07, *t*(248) = 8.48, *p* < 0.001, 95% CI [0.43, 0.70], and gender *B* = −0.43, SE = 0.11, *t*(248) = −3.85, *p* < 0.001, 95% CI [−0.65, −0.21]. Agentic Extraversion did not predict animal-centered empathy, *B* = 0.03, SE = 0.06, *t*(248) = 0.44, *p* = 0.661, 95% CI [−0.09, 0.14]. As expected, Antagonism negatively predicted animal-centered empathy, *B* = −0.14, SE = 0.06, *t*(248) = −2.27, *p* = 0.024, 95% CI [−0.26, −0.02], and Narcissistic Neuroticism positively predicted animal-centered empathy, *B* = 0.15, SE = 0.05, *t*(248) = 2.81, *p* = 0.005, 95% CI [0.04, 0.25].

### Narcissism and attitude towards animals and animal attachment

We used Agentic Extraversion, Antagonism, and Narcissistic Neuroticism to predict attitudes towards animals, controlling for gender (for the full regression table, see Supplementary Materials: Supplementary Table 1). Agentic Extraversion did not predict attitudes towards animals, *B* = 0.06, SE = 0.07, *t*(249) = 0.97, *p* = 0.332, 95% CI [−0.07, 0.19]. Antagonism negatively predicted attitudes towards animals, *B* = −0.25, SE = 0.06, *t*(249) = −3.90, *p* < 0.001, 95% CI [−0.37, −0.12], and Narcissistic Neuroticism positively predicted attitudes towards animals, *B* = 0.23, SE = 0.06, *t*(249) = 3.93, *p* < 0.001, 95% CI [0.11, 0.35].

Next, we conducted a series of regressions using Agentic Extraversion, Antagonism, and Narcissistic Neuroticism to predict each subscale measuring pet attachment, while also controlling for gender (see Supplementary Materials: Supplementary Table 1). Antagonism predicted more negative impact from owning a pet, *B* = −0.15, SE = 0.07, *t*(249) = −2.13, *p* = 0.034, 95% CI [−0.30, −0.01], but Agentic Extraversion and Narcissistic Neuroticism did not, all Bs ≤ 0.08, SE*s* ≤ 0.07, *ts*(249) ≤ 1.21, *ps ≥ 0*.226.

Narcissistic Neuroticism predicted greater emotion regulation from owning a pet, *B* = 0.15, SE = 0.06, *t*(249) = 2.36, *p* = 0.019, 95% CI [0.02, 0.27], but Agentic Extraversion and Antagonism did not, all Bs ≤ 0.12, SE*s* ≤ 0.07, *ts*(249) ≤ 1.79, *ps ≥ 0*.075.

Agentic Extraversion, Antagonism, and Narcissistic Neuroticism did not predict the extent to which someone indicated loving their pet, all Bs ≤ 0.04, SE*s* ≤ 0.06, *ts*(249) ≤ 1.54, *ps ≥ 0*.124, or personal growth from owning a pet, all Bs ≤ 0.09, SE*s* ≤ 0.07, *ts*(249) ≤ 1.33, *ps ≥ 0*.186.

## Discussion

Although it is widely thought that empathy for humans corresponds to empathy for animals (Eisenberg, 1988), researchers have demonstrated that people’s empathy for animals is distinct from their empathy for humans (Paul, 2000; Gómez-Leal et al., 2021). Lower human-centered empathy is commonly associated with narcissistic characteristics, but little is known about the animal-centered empathy in pet owners who are high (vs. low) in narcissism. The goal of this study was to examine the relation between three aspects of narcissism (i.e., Agentic Extraversion, Narcissistic Neuroticism, and Antagonism) and human- and animal-centered empathy, attitudes towards animals, and pet attachment among pet owners. We found that individuals with certain narcissistic traits display different levels of empathy for animals and humans.

We did not find evidence of an association between Agentic Extraversion and human-centered empathy, animal-centered empathy, pet attachment, or attitudes toward animals. Thus, pet owner’s overinflated, grandiose self-views do not seem to relate to the way in which they empathize with or build attachment to their pets. This is notable because previous research has largely focused on grandiose narcissism using assessments that combine Agentic Extraversion and Antagonism (e.g., Vonk et al., 2016). Using the three-factor approach to studying narcissistic traits allows us to clarify the limited role of Agentic Extraversion in pet owner’s tendencies to be empathic toward, and attached to, their pet.

Antagonism was related to experiencing less empathy for both humans and animals, though this relation is stronger for human-centered empathy. This is consistent with previous research in humans where there is typically a robust and negative relation between Antagonism (i.e., narcissistic rivalry) and empathy (Back et al., 2013). Antagonism also predicted more negative attitudes toward animals and experiencing more negative outcomes from owning a pet. These results replicate past findings where researchers suggest that narcissistic antagonism is primarily related to more negative attitudes towards animals (Kavanagh et al., 2013). Thus, individuals high in Antagonism may have more pathological and antisocial motives for their empathy deficit that extends to animals as well as humans.

Narcissistic Neuroticism was unrelated to human-centered empathy but positively related to animal-centered empathy. We also found that individuals high in Narcissistic Neuroticism reported more positive attitudes towards animals and reported experiencing greater emotional regulation from a pet, though they also reported experiencing more negative impacts of being a pet owner. Individuals high in Narcissistic Neuroticism may empathize with animals to a greater extent than they empathize with humans and may use their pet as a means of regulating their negative emotions and controlling their insecurities. Individuals possessing characteristics of vulnerable narcissism (i.e., strong feelings of social disconnect and interpersonal sensitivity) are known to struggle emotionally because of their fear of rejection and social isolation (Miller et al., 2011). Pets can be used to form a social connection with another living being and avoid feelings of social rejection (Staats et al., 2008; McConnell et al., 2011; Paul et al., 2014). As well, this effect may be modulated by the growing body of research that shows the human-animal bond may be significantly driven by the animal’s capacity to provide non-judgmental social support (Kruger and Serpell, 2010; Fine and Beck, 2015; Fine and Weaver, 2018). This, combined with the fact that pet animals are perceived as being more available to meet the emotional needs of the owner (e.g., to anthropomorphize, they are never “too busy”; McNicholas and Collis, 1995), provides a theoretical explanation as to why pet ownership may be especially important for individuals high in vulnerable narcissism.

Because the Trifurcated Model of Narcissism separates Antagonism from both Agentic Extraversion and Narcissistic Neuroticism, we were able to extend previous research on the negative role of the antagonism as it relates to a less empathy towards humans *and* animals. Moreover, exploring the three factors of narcissism separately also highlights how individuals high in Narcissistic Neuroticism may empathize with animals to a greater extent than they empathize with humans, as well as how individuals high in Antagonism demonstrate anti-social tendencies towards humans and animals. Notably, exploring the three-factor model of narcissism provides insight into the motives for people’s animal-centered empathic concern.

### Limitations and considerations for future research

Given our limited sample, there are a host of additional moderators that researchers may wish to explore. It is important to note that we preselected individuals who reported owning both a cat and a dog, so these individuals may not be representative of the general population; future research may wish to consider how animal-centered empathy varies in individuals who are and are not pet owners. Furthermore, researchers may decide to examine how the different types of pets (e.g., typical or non-typical pets) may affect levels of animal and human-centered empathy in pet owners with narcissistic traits, particularly by evaluating narcissism using the Trifurcated Model. The work of Vonk et al. (2016), which evaluated traditional and non-traditional pet owners’ levels of grandiose and vulnerable narcissism, as well as tendency towards borderline personality disorder, suggests the type of pet may be an important factor to consider. Furthermore, it is also important to explore how the Trifurcated Model of Narcissism relates to the depth of pet ownership, or the degree of responsibility people take for their pets. Notably, this research is correlational and should be interpreted cautiously. Future research should adopt experimental approaches to explore whether individuals high in Antagonism can feel greater empathy for animals (e.g., Hepper et al., 2014).

Because we used a convenience sample of undergraduate university students, it may be reasonable to question whether they were the sole owner of their pet or whether the pet belonged to their guardians. With that being said, many of our participants indicated that they were actively engaged in taking care of their pets, so this may not be a critical factor to consider in future work. It is also important to consider measurement issues. Some of our results may need to be interpreted cautiously when scale reliabilities are particularly low. For example, the negative impact subscale of the PALS was quite low, likely due to the limited number of items included in the subscale. Finally, we only examined subclinical narcissism; researchers may wish to use a target population of pet owners who are diagnosed with Narcissistic Personality Disorder when exploring animal-centered empathy in future research. In addition, we assess the three aspects of narcissism using a combination of scales. Future researchers may want to adopt the Five-Factor Narcissism Inventory (Glover et al., 2012), which assesses the Trifurcated Model of Narcissism in a single measure. These suggestions can help guide future research to further investigate the differences in animal and human-centered empathy in narcissistic individuals.

### Relevance of the research

From a human-animal interaction perspective, this research highlights another way in which human-centered empathy may be related to, but distinct from, animal centered-empathy. Research on animal-centered empathy is important, as it could provide further insight into empathy in narcissistic individuals. Narcissistic individuals can be empathic towards others when they are encouraged to take the perspective of the person in need (Hepper et al., 2014). Some therapeutic approaches for Narcissistic Personality Disorder involve teaching individuals how empathy looks and when it should be used, which assumes that these individuals have little sense of empathy (Baskin-Sommers et al., 2014). Researchers have previously found that having cats and dogs who are dependent on humans can help individuals practice caring about animals (Rothgerber and Mican, 2014) and pet ownership has been found to be associated with higher prosocial orientation (Vidović et al., 1999). We found that pet owners who are higher in vulnerable narcissism often use their pet as a way of regulating their emotions and finding comfort, which may have implications for therapy that deals with managing complications associated with vulnerable narcissism. For instance, animal-assisted therapy may be used in the management of individuals who are high in vulnerable narcissism. These individuals may benefit from being around animals, as our study demonstrates that owning a pet helps pet owners with vulnerable narcissistic traits regulate their emotions.

There are various anecdotes throughout history that demonstrate how animal and human-centered empathy are different constructs. For instance, militant animal rights activists have shown little human-centered empathy, such that they have threatened and injured people while they display concern for animal welfare (Paton, 1993). This example illustrates that individuals who are violent towards humans and may have little human-centered empathy can still retain a high sense of animal-centered empathy. Here, we explored how individuals with narcissistic traits may experience different levels of animal-centered and human-centered empathy. Although empathy for animals and humans is certainly related, it is possible for pet owners with narcissistic traits, particularly Narcissistic Neuroticism, to have empathy and love for a pet despite having low empathy for humans.

## Data availability statement

The raw data supporting the conclusions of this article will be made available by the authors, without undue reservation.

## Ethics statement

The studies involving human participants were reviewed and approved by MacEwan University’s Research Ethics Board. The patients/participants provided their written informed consent to participate in this study.

## Author contributions

MG, EJ, and EL contributed to conception and design of the study. EJ programmed survey and collected data. MG performed statistical analyses and MG and EL wrote manuscript. All authors contributed to manuscript revision, read, and approved the submitted version.

## Funding

Funding is provided by the MacEwan University Office of Research Services.

## Conflict of interest

The authors declare that the research was conducted in the absence of any commercial or financial relationships that could be construed as a potential conflict of interest.

## Publisher’s note

All claims expressed in this article are solely those of the authors and do not necessarily represent those of their affiliated organizations, or those of the publisher, the editors and the reviewers. Any product that may be evaluated in this article, or claim that may be made by its manufacturer, is not guaranteed or endorsed by the publisher.

## Supplementary material

The Supplementary material for this article can be found online at: https://www.frontiersin.org/articles/10.3389/fpsyg.2023.1087049/full#supplementary-material

## Footnotes

^1^We focus on narcissism as a subclinical personality trait, rather than Narcissistic Personality Disorder. Here, we measure narcissism continuously and recognize that there is no evidence for a class of "narcissists" (Aslinger et al., 2018).

^2^ https://osf.io/g3fa5/
