# Supplementary material for: Exploring narcissism and human- and animal-centered empathy in pet owners
Source: Front Psychol. 2023 Mar 30;14:1087049. doi: 10.3389/fpsyg.2023.1087049 (PMC10098159; doi:10.3389/fpsyg.2023.1087049)
Supplement: Supplementary file 1 [file Table_1.pdf]

Supplemental Materials

Supplemental Table 1

Regression analyses using narcissistic traits to predict human- and animal-centered empathy, attitude towards animals, and pet attachment.

Predictors	*B*	*SE*	*t*	*p*	95% CI
**Dependent variable: Human-centered Empathy**					
Agentic Extraversion	.03	.05	0.58	.584	[-.07, .12]
Antagonism	-.19	.05	-3.77	**<.001**	[-.28, -.09]
Narcissistic Neuroticism	.01	.05	0.15	.879	[-.08, .10]
Gender	-.18	.10	-1.86	.064	[-.37, .01]
Animal-centered empathy	.40	.05	8.48	**<.001**	[.31, .49]
**Dependent variable: Animal-centered Empathy**					
Agentic Extraversion	.03	.06	0.44	.661	[-.09, .14]
Antagonism	-.14	.06	-2.27	**.024**	[-.26, -.02]
Narcissistic Neuroticism	.15	.05	2.81	**.005**	[.05, .25]
Gender	-.43	.11	-3.85	**<.001**	[-.65, -.21]
Human-centered empathy	.56	.07	8.48	**<.001**	[.43, .70]
**Dependent variable: Attitudes toward Animals**					
Agentic Extraversion	.06	.07	0.97	.332	[-.07, .19]
Antagonism	-.25	.06	-3.90	**<.001**	[-.37, -.12]
Narcissistic Neuroticism	.23	.06	3.93	**<.001**	[.12, .35]
Gender	-.59	.12	-4.86	**<.001**	[-.83, -.35]
**Dependent variable: Emotion Regulation – Pet Attachment**					
Agentic Extraversion	.04	.07	0.60	.550	[-.10, .18]
Antagonism	-.12	.07	-1.79	.075	[-.26, .01]
Narcissistic Neuroticism	.15	.06	2.36	**.019**	[.02, .27]
Gender	-.68	.13	-5.26	**<.001**	[-.94, -.43]
**Dependent variable: Love – Pet Attachment**					
Agentic Extraversion	.09	.06	1.38	.169	[-.04, .21]
Antagonism	-.09	.06	-1.54	.124	[-.21, .03]
Narcissistic Neuroticism	.04	.06	.77	.443	[-.07, .15]
Gender	-.70	.12	-6.10	**<.001**	[-.92, -.47]
**Dependent variable: Negative Impact – Pet Attachment**					
Agentic Extraversion	.00	.07	-.01	.996	[-.15, .14]
Antagonism	-.15	.07	-2.13	**.034**	[-.30, -.01]
Narcissistic Neuroticism	-.08	.07	-1.21	.226	[-.21, .05]

Gender	.09	.14	0.65	.517	[-.18, .36]
**Dependent variable: Personal Growth – Pet Attachment**					
Agentic Extraversion	.02	.07	0.23	.819	[-.13, .16]
Antagonism	-.07	.07	-1.06	.292	[-.21, .06]
Narcissistic Neuroticism	-.09	.07	1.32	.186	[-.04, .22]
Gender	-.32	.13	-2.36	**.019**	[-.58, -.05]

Note. All variables were standardized as z-scores. Gender is coded 1 = male, 0 = female.
